# Risk of active tuberculosis among COPD patients treated with fixed combinations of long-acting beta2 agonists and inhaled corticosteroids

**DOI:** 10.1186/s12879-020-05440-6

**Published:** 2020-09-25

**Authors:** Tsan-Ming Huang, Kuan-Chih Kuo, Ya-Hui Wang, Cheng-Yi Wang, Chih-Cheng Lai, Hao-Chien Wang, Likwang Chen, Chong-Jen Yu, On the behalf of Taiwan Clinical Trial Consortium for Respiratory Diseases (TCORE), Chong-Jen Yu, Hao-Chien Wang, Diahn-Warng Perng, Shih-Lung Cheng, Jeng-Yuan Hsu, Wu-Huei Hsu, Jeng-Yuan Hsu, Wu-Huei Hsu, Ying-Huang Tsai, Tzuen-Ren Hsiue, Meng-Chih Lin, Hen-I Lin, Cheng-Yi Wang, Yeun-Chung Chang, Ueng-Cheng Yang, Cing-Syong Lin, Likwang Chen, Yu-Feng Wei, Inn-Wen Chong, Chung-Yu Chen

**Affiliations:** 1 Department of Internal Medicine, Cardinal Tien Hospital and School of Medicine, College of Medicine, Fu Jen Catholic University, New Taipei City, Taiwan; 2 Division of Pulmonary, Department of Internal Medicine, MacKay Memorial Hospital, Taipei, Taiwan; 3 Medical Research Center, Cardinal Tien Hospital and School of Medicine, College of Medicine, Fu Jen Catholic University, New Taipei City, Taiwan; 4 Department of Internal Medicine, Kaohsiung Veterans General Hospital, Tainan Branch, Tainan, Taiwan; 5 Department of Internal Medicine, National Taiwan University Hospital and College of Medicine, National Taiwan University, Taipei, Taiwan; 6 Institute of Population Health Sciences, National Health Research Institutes, Zhunan, Miaoli County Taiwan

**Keywords:** Fluticasone/salmeterol, Budesonide/formoterol, COPD, Tuberculosis

## Abstract

**Objectives:**

To investigate the incidence of active tuberculosis (TB) among COPD patients using fluticasone/salmeterol or budesonide/formoterol, and to identify any differences between these two groups of patients.

**Methods:**

The study enrolled COPD patients from Taiwan NHIRD who received treatment with fluticasone/salmeterol or budesonide/formoterol for > 90 days between 2004 and 2011. The incidence of active TB was the primary outcome.

**Results:**

Among the intention-to-treat population prior to matching, the incidence rates of active TB were 0.94 and 0.61% in the fluticasone/salmeterol and budesonide/formoterol groups, respectively. After matching, the fluticasone/salmeterol group had significantly higher rates of active TB (adjusted HR, 1.41, 95% CI, 1.17–1.70) compared with the budesonide/formoterol group. The significant difference between these two groups remained after a competing risk analysis (HR, 1.45, 95% CI, 1.21–1.74). Following propensity score matching, the fluticasone/salmeterol group had significantly higher rates of active TB compared with the budesonide/formoterol group (adjusted HR, 1.45, 95% CI, 1.14–1.85). A similar trend was observed after a competing risk analysis (HR, 1.44, 95% CI, 1.19–1.75). A higher risk of active TB was observed in the fluticasone/salmeterol group compared with the budesonide/formoterol group across all subgroups, but some differences did not reach statistical significance.

**Conclusion:**

Fluticasone/salmeterol carried a higher risk of active TB compared with budesonide/formoterol among COPD patients.

## Introduction

Tuberculosis (TB) is a serious public health problem in many countries, including Taiwan [1–6]. In addition to the use of appropriate anti-TB treatment, early detection in vulnerable populations is an important way of preventing its spread [7]. Many well-known risk factors, including HIV infection, diabetes mellitus, socioeconomic status, alcoholism and immunocompromised condition have been reported as being significantly associated with TB [8–10]. In addition to these risk factors, many studies have shown that the use of corticosteroids, in both systemic and inhaled forms, can also increase a patient’s risk of TB [11–15]. Regarding the association between inhaled corticosteroid (ICS) and TB, one meta-analysis of 25 trials by Dong et al. showed ICS treatment was associated with a significantly higher risk of TB (Peto OR, 2.29; 95% CI, 1.04–5.03, 14) and another meta-analysis of nine non-randomized studies by Castellana et al. showed that any ICS use was associated with an increased risk of TB versus no ICS use (OR = 1.46; 95% CI 1.06 to 2.01). and a similar trend was also found for current ICS use versus prior/no ICS use, as well as for high, moderate and low ICS dose versus no ICS [15].

However, ICS are a major treatment component for patients with chronic obstructive pulmonary disease (COPD), and the combination of inhaled long-acting β2-agonists (LABAs) and ICSs has been shown to be effective for the reduction of COPD exacerbations [16–18]. Although many LABA/ICS combinations have been developed, clinical experience using budesonide/formoterol and fluticasone/salmeterol, as well as associated studies, are most plentiful because these two combinations were developed earliest. Recently, several studies have revealed safety differences between fluticasone/salmeterol and budesonide/formoterol [19–21]. These studies found that fluticasone/salmeterol users have a higher risk of pneumonia, sepsis, and death compared with budesonide/formoterol users [19–21]. However, no study has previously compared the risk of TB between fluticasone/salmeterol and budesonide/formoterol. Therefore, the current study investigated the incidence of active TB among COPD patients using fluticasone/salmeterol and budesonide/formoterol, and to see if there were any differences between these two groups.

## Methods

### Data source

The current study used a subset of the Taiwan National Health Insurance Research Database (NHIRD), which contained information on 2,200,000 individuals with heart or lung disease. Because the records of patients have been anonymized and de-identified, no informed consent was required. Ethical approval was obtained from the Institutional Review Board of Cardinal Tien hospital (No. CTH 108–3–5-013).

### Patients selection

**Fig. 1:**
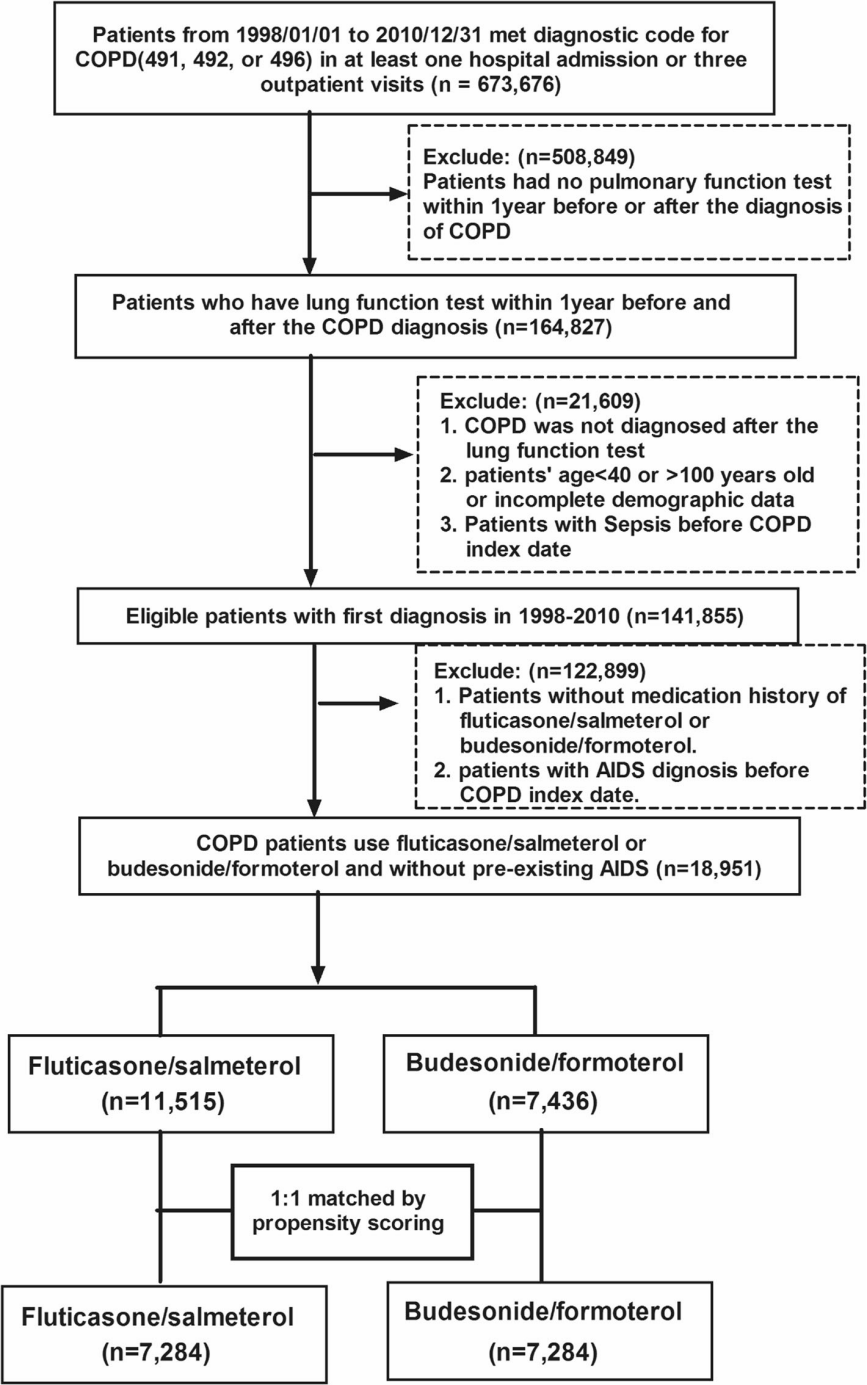
Study algorithm

COPD patients aged 40 to 100 years were identified using International Classification of Disease, Ninth Revision (ICD-9)-CM codes 491, 492 and 496 according to previous study [20]. The present study consisted of COPD patients who received treatment with fluticasone/salmeterol or budesonide/formoterol for > 90 days between 2004 and 2011. Patients with a reported history of AIDS were excluded (*n* = 5). Patients were followed until 31 December 2011, the end of the fixed combination treatment, emigration or death, whichever came first. Figure 1 describes the study population selection process.

### Measurement of outcome

The case of active TB cases was defined using the ICD-9 codes (010–018, including all subcategories) and based on the use of at least 28 days anti-TB drug as previous studies [12, 22].

### Definition of exposure and confounding factor

We defined fixed LABA/ICS combinations using Anatomical Therapeutic Chemical (ATC) codes R03AK06 or R03AK07, according to previously reported [20]. We only calculated the event of COPD exacerbation during the usage of same fixed LABA/ICS combination period. In contrast, once the patient changed to the other fixed combination, the case was censored. We also collected the data regarding the use of ICSs (ATC code R03BA), LABAs (ATC codes R03AC12 and R03AC13), short-acting β2-agonists (ATC code R03AC) and other related drugs.

### Statistical analysis

To reduce potential confounding caused by unbalanced covariates, we used the pairwise 1:1 propensity score matching and logistic regression to construct two comparable groups. We start the process with the smallest group (the budesonide/formoterol group 7436 patients) and matches them 1:1 to the larger treatment population (the fluticasone/salmeterol group). After the matching process, the two groups were no differences in the underlie characteristics including: age, sex, year of index date, monthly income, hospital level, COPD medications, comorbidities and the episodes of previous severe COPD exacerbations (emergency department visits or COPD-related hospitalizations).

We conduct both Intention-to-treat (ITT) and as-treated (AT) analyses to see the effects. ITT analyses ignore noncompliance, drug switching, subsequent withdrawal or deviation after their original treatment allocation. In AT analyses, the patients were censored on the day of medication switching, add-on or discontinuation.

The crude and adjusted hazard ratios (HRs) of active TB between the two study groups were calculated by Cox regression models with adjustment for age, gender and propensity scores. A *P* value of < 0.05 indicated statistical significance in all analyses. The analyses were conducted using SAS software version 9.4 (SAS Institute Inc., Cary, NC, USA).

## Results

### Patient characteristics

Initially, 18,951 patients received a fixed LABA/ICS combination (11,515 received fluticasone/salmeterol and 7436 received budesonide/formoterol). Before propensity score matching, the fluticasone/salmeterol group were older, more likely to be male, had higher Charlson scores and more comorbidities (including myocardial infarction, congestive heart failure, cerebrovascular disease, dementia, diabetes, and malignancy) than the budesonide/formoterol group (Table 1). In addition, the fluticasone/salmeterol group had a lower income, more episodes of COPD exacerbations and less use of COPD inhaled and oral drugs (except LAMA), than the budesonide/formoterol group. Further pairwise matching (1:1) of fluticasone/salmeterol and budesonide/formoterol group identified two similar subgroups each comprising 7284 cases (Table 1).

**Table 1:** Baseline characteristics of Fluticasone/salmeterol and budesonide/formoterol cohort before and after matching

Variables	Before PS matching					After PS matching				
	Fluticasone/salmeterol cohort (*n* = 11,515)		Budesonide/formoterol cohort (*n* = 7436)		*p* value	Fluticasone/salmeterol cohort (*n* = 7284)		Budesonide/formoterol cohort (n = 7284)		*p* value
	n	(%)	n	(%)		n	(%)	n	(%)	
**Index year**					<.0001					0.7872
2004	1963	(17.04)	1430	(19.23)		1434	(19.69)	1407	(19.32)	
2005	1261	(10.95)	1230	(16.54)		1118	(15.35)	1138	(15.62)	
2006	1326	(11.51)	990	(13.31)		976	(13.4)	970	(13.32)	
2007	1485	(12.89)	973	(13.08)		1002	(13.76)	967	(13.28)	
2008	1375	(11.94)	843	(11.34)		835	(11.46)	833	(11.44)	
2009	1542	(13.39)	762	(10.25)		749	(10.28)	761	(10.45)	
2010	1570	(13.63)	815	(10.96)		822	(11.29)	815	(11.19)	
2011	993	(8.62)	393	(5.28)		348	(4.78)	393	(5.40)	
**Age (year)**	65.95 ± 10.26		63.29 ± 10.40		<.0001	63.77 ± 10.35		63.53 ± 10.31		0.1535
**Male Gender**	8798	(76.38)	5433	(73.05)	<.0001	5403	(74.18)	5348	(73.42)	0.3001
**Monthly income**					0.0007					0.9766
< 19,100	4037	(35.05)	2481	(33.36)		2443	(33.54)	2451	(33.65)	
19,100-41,999	6011	(52.19)	3874	(52.09)		3812	(52.33)	3799	(52.16)	
≧42,000	1467	(12.74)	1081	(14.54)		1029	(14.13)	1034	(14.20)	
**Hospital level**					<.0001					0.9562
Level 1	4649	(40.36)	3076	(41.36)		2970	(40.77)	3001	(41.20)	
Level 2	4886	(42.42)	2907	(39.09)		2893	(39.72)	2877	(39.50)	
Level 3	1476	(12.81)	1016	(13.66)		997	(13.69)	991	(13.61)	
Level 4 (rural area)	504	(4.38)	437	(5.88)		424	(5.82)	415	(5.70)	
**COPD medications**										
Oral steroids	4544	(39.45)	2691	(36.18)	<.0001	2683	(36.83)	2660	(36.52)	0.6925
Antibiotics	8142	(70.69)	5344	(71.86)	0.0855	5202	(71.42)	5227	(71.76)	0.6460
LABA	412	(3.58)	366	(4.92)	<.0001	336	(4.61)	347	(4.76)	0.6664
SABA	3400	(29.52)	2514	(33.80)	<.0001	2419	(33.21)	2434	(33.42)	0.7920
LAMA	1488	(12.92)	711	(9.56)	<.0001	701	(9.62)	711	(9.76)	0.7794
Theophylline	8144	(70.71)	5378	(72.31)	0.0175	5248	(72.05)	5257	(72.17)	0.8679
Aminophylline	4884	(42.40)	3211	(43.18)	0.2969	3145	(43.18)	3142	(43.14)	0.9600
ICS	3155	(27.39)	2591	(34.84)	<.0001	2465	(33.84)	2478	(34.02)	0.8200
**Severe AE**					<.0001					0.9873
0	6339	(55.04)	4579	(61.57)		4459	(61.22)	4451	(61.11)	
1	1914	(16.62)	1102	(14.82)		1090	(14.96)	1090	(14.96)	
2+	3262	(28.32)	1755	(23.60)		1735	(23.82)	1743	(23.93)	
***Baseline Comorbidities***										
Charlson Score	1.64 ± 1.00		1.55 ± 0.90		<.0001	1.55 ± 0.91		1.55 ± 0.90		0.7699
Myocardial infarction	190	(1.65)	95	(1.28)	0.0397	97	(1.33)	95	(1.30)	0.8845
Congestive heart failure	1030	(8.94)	584	(7.85)	0.0086	566	(7.77)	579	(7.95)	0.6890
Peripheral vascular disease	92	(0.80)	52	(0.70)	0.4405	52	(0.71)	51	(0.70)	0.9212
Cerebrovascular disease	578	(5.02)	279	(3.75)	<.0001	277	(3.80)	277	(3.80)	1.0000
Dementia	193	(1.68)	67	(0.90)	<.0001	66	(0.91)	67	(0.92)	0.9306
Rheumatologic disease	114	(0.99)	74	(1.00)	0.9722	67	(0.92)	71	(0.97)	0.7323
Peptic ulcer disease	1702	(14.78)	1052	(14.15)	0.2271	1032	(14.17)	1030	(14.14)	0.9621
Hemiplegia or paraplegia	7	(0.06)	2	(0.03)	0.2957	3	(0.04)	2	(0.03)	0.6547
Renal disease	270	(2.34)	153	(2.06)	0.1913	149	(2.05)	146	(2.00)	0.8599
Diabetes	1253	(10.88)	706	(9.49)	0.0022	690	(9.47)	703	(9.65)	0.7142
Moderate or severe liver disease	374	(3.25)	239	(3.21)	0.8977	229	(3.14)	235	(3.23)	0.7771
Tumor	372	(3.23)	202	(2.72)	0.0438	201	(2.76)	199	(2.73)	0.9192
Autoimmune	660	(5.73)	442	(5.94)	0.5418	445	(6.11)	428	(5.88)	0.5529

### Risk of incidental active TB

Among the intention-to-treat population prior to matching, the incidence rates of active TB were 0.94 and 0.61% in the fluticasone/salmeterol and budesonide/formoterol groups, respectively. The fluticasone/salmeterol group had significantly higher rates of active TB (adjusted HR, 1.41, 95% CI, 1.17–1.70) than the budesonide/formoterol group (Table 2). The significant difference between these two groups remained after competing risk analysis (HR, 1.45, 95% CI, 1.21–1.74). Even following propensity score matching, the fluticasone/salmeterol group had a significantly higher rate of active TB compared with the budesonide/formoterol group (adjusted HR, 1.45, 95% CI, 1.14–1.85), and this was maintained after competing risk analysis (HR, 1.44, 95% CI, 1.19–1.75). Furthermore, the higher risk of active TB among the fluticasone/salmeterol group compared with the budesonide/formoterol group remained in the as-treated population analysis (Table 2) but the difference was not significant.

**Table 2:** Incidence rates, hazard ratios and competing risk of active TB associated with Fluticasone/salmeterol relative to Budesonide/formoterol in patients with COPD in intent-to-treat and as treated analysis

Fluticasone/salmeterol cohort				Budesonide/formoterol cohort			Crude HR (95%CI)	Adjusted^b^ HR (95%CI)	Competing risk subHR (95%CI)
event		Person-Year	IR^a^	event	Person-Year	IR^a^			
**ITT analysis**									
Before propensity score matching									
TB	358	38,123.37	0.94%	175	28,559.41	0.61%	1.50 (1.26–1.80)	1.41 (1.17–1.70)	1.45 (1.21–1.74)
After propensity score matching									
TB	247	27,392.73	0.90%	172	27,689.85	0.62%	1.34 (1.07–1.69)	1.45 (1.14–1.85)	1.44 (1.19–1.75)
**As treated analysis**									
Before propensity score matching									
TB	126	14,256.75	0.88%	66	10,120.93	0.65%	1.32 (0.98–1.78)	1.28 (0.94–1.73)	1.27 (0.94–1.71)
After propensity score matching									
TB	85	9512.25	0.89%	66	9872.11	0.67%	1.23 (0.73–2.07)	1.38 (0.79–2.42)	1.31 (0.95–1.81)

^a^*IR* Incidence rate

^b^Adjusted for age, gender, propensity score

### Subgroup analysis

**Fig. 2:**
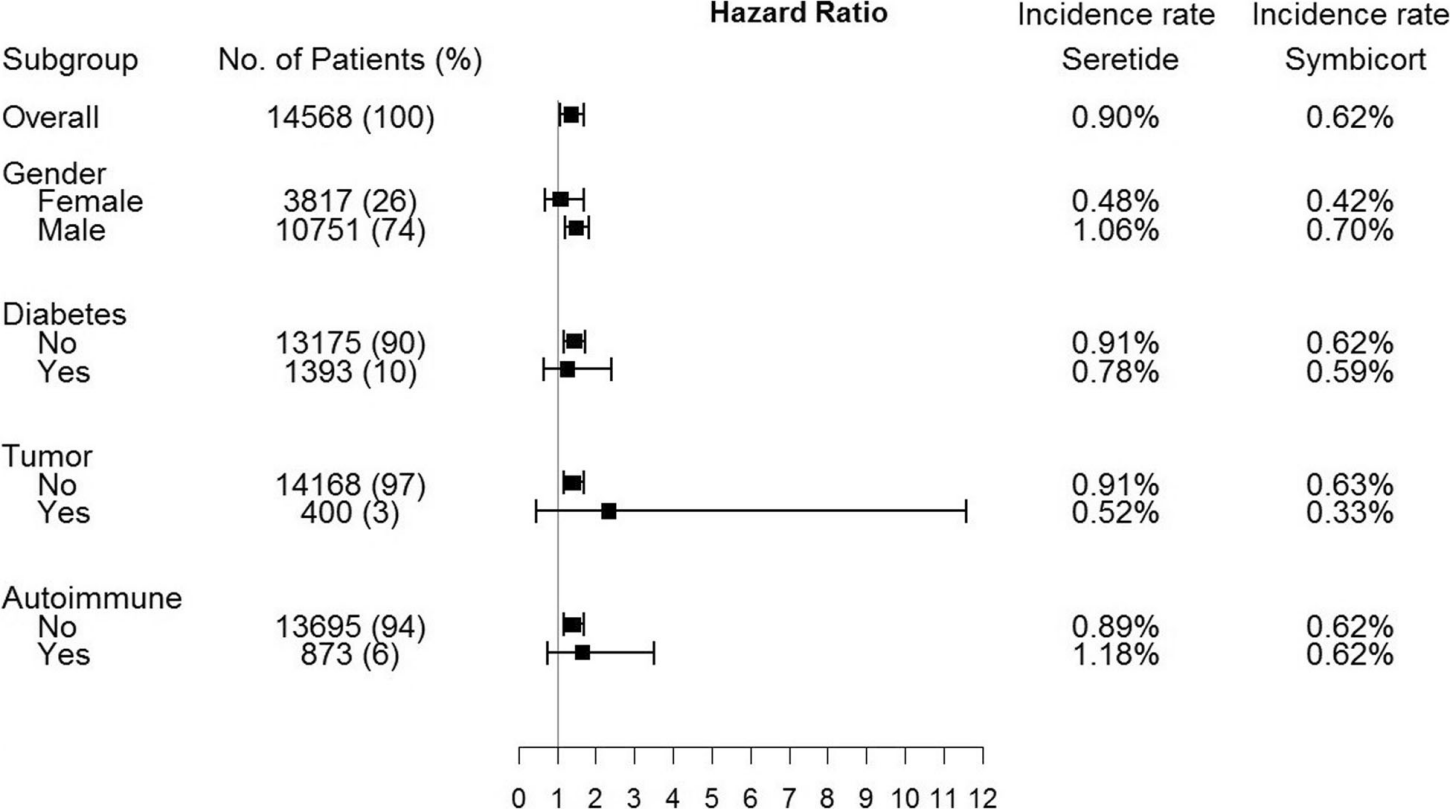
Subgroup analysisTable 1 Baseline characteristics of Fluticasone/salmeterol and budesonide/formoterol cohort before and after matching

A subgroup analysis was performed using an intention-to-treat analysis. A higher incidence of active TB was observed in the fluticasone/salmeterol group compared with the budesonide/formoterol group across all subgroups (male patients, 1.06% vs 0.70%; patients without diabetes mellitus, 0.91% vs 0.62%; patients without cancer, 0.91% vs 0.63%; and patients without autoimmune disease, 0.89% vs 0.62%; all *p* < 0.05), but some differences did not reach statistical significance (female patients, 0.48% vs 0.42%; patients with diabetes mellitus, 0.78% vs 0.59%; patients with cancer, 0.52% vs 0.33%; and patients with autoimmune disease, 1.18% vs 0.62%) (Fig. 2).

## Discussion

The present national population-based study demonstrated that COPD patients receiving fluticasone/salmeterol had a higher risk of active TB compared with those receiving budesonide/formoterol. In contrast to previous meta-analyses [14, 15] showed that ICS increased the risk of tuberculosis, this study is the first one to point out patients using fluticasone/salmeterol are at higher risk of tuberculosis then patients using budesonide/formoterol. This significant difference between budesonide/formoterol and fluticasone/salmeterol was confirmed in various populations, including intention-to-treat and after propensity score matching methods and competing risk analysis. This trend was also observed in the as-treated population with nearly the same proportion using different methods. However, the non-significant difference could be due to the low case number in as treated analysis. Even in the subgroup analysis, the fluticasone/salmeterol group remained associated with a higher risk of active TB compared with the budesonide/formoterol group. This intra-class difference has been shown in a previous study [21], and there may be different risks for other infectious diseases between budesonide/formoterol and fluticasone/salmeterol. In the PATHOS study, the rate of pneumonia and the pneumonia event rate per 100 patients were higher in fluticasone/salmeterol compared with budesonide/formoterol [19]. Another study showed that fluticasone/salmeterol carried a higher risk of sepsis (aHR, 1.15; 95%CI, 1.07–1.24) and septic shock (aHR, 1.14; 95%CI, 1.01–1.29) compared with budesonide/formoterol [21]. However, the present study is the first to confirm an intra-class difference for the risk of active TB between fluticasone/salmeterol and budesonide/formoterol. Therefore, these findings confirmed the significant differences between budesonide/formoterol and fluticasone/salmeterol in terms of the risk of infectious diseases, including TB, in patients with COPD.

As this study was an observational cohort study, it was not possible to investigate the possible mechanisms that caused the differences between fluticasone and budesonide. However, several different pharmacologic characteristics between these two agents may give some explanations. First, the uptake and elimination rates are slower for fluticasone than budesonide, as reported in a previous pharmacokinetic study [23]. The mean residence time of budesonide was shorter than fluticasone, and the amount of expectorated fluticasone was significantly higher compared with budesonide [24]. Second, fluticasone and budesonide also differ in the immune response that they influence. In vitro studies have shown that fluticasone is about 10 times more potent than budesonide in inhibiting the release of IL-6, IL-8, and TNF-α production [25]. Third, budesonide is less lipophilic than fluticasone. In summary, all of these factors suggest that fluticasone can reside in humans for longer and could cause a more potent immunosuppressive effect than budesonide, thereby facilitating TB infection.

The present study had two major strengths. First, it was conducted using data from the NHIRD database, which includes nearly all patients in Taiwan. Using this database, it is possible to enroll a large population and obtain long-term follow-up data for the included subjects. Therefore, the findings can be considered representative and truly reflective of the real-world. Second, TB remains prevalent in Taiwan, and the incidence of TB in Taiwan is much higher than in Western countries. Therefore, the cohort contained a large number of TB cases, which can help increase the statistical power of the analysis for subgroup and confounding factor adjustment. Nevertheless, the current study had several limitations. First, although many variables were collected to minimize the confounding effect, the effect of some potential confounder, such as BMI, pulmonary function, the status of smoking and history of latent TB, data for which was not available in the NHIRD database. However, the status of smoking and latent TB could have significant impact on the development of active TB. Further study is warranted to assess their effect on the risk of TB. Second, microbiological data is lacking in the NHIRD database, however, this data is the key diagnostic criteria for active TB. To overcome potential misclassification of TB in the claims database, cases of active TB were only identified when they had concurrent ICD-9 codes for TB and a history of anti-TB medication. Third, the effect of dose and duration of LABA/ICS was not assessed in this study.

## Conclusions

Fluticasone/salmeterol carried a higher risk of active TB than budesonide/formoterol among COPD patients.

## Data Availability

The original databases used and/or analyzed during the current study, which were released and permitted by the National Health Research Institutes, are closed for releasing now. The working datasets for statistical analysis in the current study are available from the corresponding author on reasonable request.

## Abbreviations

COPD: Chronic obstructive pulmonary disease;
TB: Tuberculosis
NHIRD: National Health Insurance Research Database
ICS: Inhaled corticosteroid
LABA: Long-acting β2-agonists
ATC: Anatomical Therapeutic Chemical
